# Green Synthesis of Nanomaterials

**DOI:** 10.3390/nano9091275

**Published:** 2019-09-07

**Authors:** Giovanni Benelli

**Affiliations:** Department of Agriculture, Food and Environment, University of Pisa, via del Borghetto 80, 56124 Pisa, Italy; giovanni.benelli@unipi.it; Tel.: +39-0502216141

Nanomaterials possess stunning physical and chemical properties. They have a major role in the development of novel and effective drugs, catalysts, sensors, and pesticides. The synthesis of nanomaterials is usually achieved via chemical and physical methods, both of which require the use of extremely toxic chemicals or high-energy inputs. To move towards more environmentally friendly processes, researchers have recently focused on so-called “green synthesis”, in which microbial, animal-, and plant-borne compounds can be used both as economic forms of waste reduction and stabilizing agents to fabricate nanomaterials. Green synthesis routes have been proposed as cheap and environmentally sustainable; they can lead to the fabrication of nano-objects with controlled size and shape, two key features affecting their bioactivity.

However, real-world applications of green-fabricated nanomaterials are still largely unexplored, and the number of marketed products is limited. One may question our knowledge about their non-target toxicity and their main modes of action. Such questions also bring up issues regarding their possible fate in the environment [1,2].

In this framework, the present Special Issue includes studies by top-ranked experts on nanosynthesis and related applications. This Special Issue includes articles on relevant and pressing issues in green nanomaterial science. Most are original research articles and all highlight theoretical concepts and practical protocols of interest for real-world applications related to nanomaterials.

Recent approaches to synthesize nanomaterials have focused on the use of various natural products. For example, healthy and microwave-injured bacteria have been used to produce finely characterized palladium nanoparticles [3]. Furthermore, plant-borne products have been employed to produce interesting nanomaterials, such as chocolate extract-fabricated Au nanoparticles with good biocompatibility features [4] as well as gum kondagogu-synthesized anatase TiO_2_ nanoparticles stable at high temperatures, which are relevant for the photocatalytic degradation of organic dyes [5]. Other nanomaterials produced include ZnO nanoparticles, which have been reduced and stabilized using the *Scadoxus multiflorus* leaf aqueous extract. These nanoparticles showed relevant antifungal and insecticidal activity and are highly effective against young instars (eggs and larvae) of *Aedes aegypti* (Diptera: Culicidae) [6], an important dengue and Zika virus mosquito vector [7].

Other valuable applications ranged from enzyme technology to biocatalysis; efforts to develop biocompatible antimicrobial surfaces have been studied by Aflori et al. [8], relying to the employ of poly-L-lactic acid. Silva et al. [9] shed light on polydopamine-mediated green reduction of graphene oxide whereas Fotiadou et al. [10] successfully designed lipase-hybrid nanoflowers, which have been enriched with carbon and magnetic nanomaterials, allowing biocatalytic transformations. Carbon nanochemistry is the focus of research by Kukulka et al. [11], which proposed a novel and reliable time-dependent facile synthesis of carbon solid spheres.

Additional studies are dedicated to other important nanomaterials, including silica-based nanomaterials. Because the granulometric characterization of silica nanomaterials requires harmonized protocols, Retamal Marin et al. [12] proposed a novel approach to investigate the impact of sample preparation on suspended nanosilica size.

Two research papers focused on the peculiarities of nanocrystals, proposing a robust protocol for the solvothermal synthesis of nanocrystalline CuInS_2_ thin films [13] and how to synthesize photofunctional mesocrystals through a polyol-based fluoride ion slow-releasing approach [14].

The Special Issue also contains a review [15] on micro- and nanoemulsions, which covers theory and practice about their preparation as well as novel applications in the fields of entomology and parasitology. A growing number of recent papers have stressed the important advantages arising from the use of green micro- and nanoemulsions to enhance the efficacy and stability of selected bioactive compounds of natural origin. Micro- and nanoemulsions of selected natural products have been successfully proposed for the management of parasites and vectors of interest for public health (e.g., mosquitoes and ticks) as well as the control of insect and mite species of agricultural importance. In the final section, the review also highlights challenges and constraints arising from the use of green micro- and nanoemulsions to promote their commercial development for various biological and biomedical purposes [15].

Overall, as the Editor of *Nanomaterials*, I am fully aware that the present Special Issue cannot fully reflect the high diversity and creativity of new concepts and tools rapidly developing in this multidisciplinary research field. However, I am confident that this focus on the green synthesis of nanomaterials will contribute to the research interest in the field, providing our readership with a multi-faceted scenario that outlines the importance of cross-field green nanoresearch, its quick growth, as well as its wide-ranging applications.

It is also expected that the present Special Issue will encourage multidisciplinary research on green nanomaterials, broadening the range of potential practical uses. This needs to be coupled with research efforts improving large-scale synthesis and economic viability of the proposed processes, ecotoxicology insights to understand the post-application fate of green nanomaterials, and their long-term stability and impact on human health and the environment.
